# Development of Super‐Paramagnetic Iron Oxide Nanoparticle‐Coated Melt Electrowritten Scaffolds for Biomedical Applications

**DOI:** 10.1002/mabi.202300397

**Published:** 2023-11-13

**Authors:** Irem Unalan, Ilenia Occhipinti, Marta Miola, Enrica Vernè, Aldo R. Boccaccini

**Affiliations:** ^1^ Institute of Biomaterials Department of Materials Science and Engineering Friedrich‐Alexander‐University Erlangen‐Nuremberg Cauerstraße 6 91058 Erlangen Germany; ^2^ Institute of Materials Physics and Engineering Department of Applied Science and Technology Politecnico di Torino Corso Duca degli Abruzzi 24 Torino 10129 Italy

**Keywords:** alkaline surface treatment, biomedical applications, magnetic nanoparticles, melt electrowriting, polycaprolactone

## Abstract

Polycaprolactone (PCL) is usually the material chosen for melt electrowriting (MEW) due to its biocompatibility, mechanical strength, and melt processability. This work first investigates the effect of different processing parameters to obtain optimum PCL‐MEW scaffolds. Secondly, to increase PCL`s hydrophilicity and cell affinity, and to enable coating with superparamagnetic iron oxide nanoparticles (SPIONs) and silica‐coated‐SPIONs (Si‐SPIONs), the scaffolds are modified with alkaline surface treatment. Finally, SPIONs and Si‐SPIONs are successfully coated on MEW scaffolds. Results show that reproducible scaffolds are fabricated. Additionally, the alkaline treatment does not change the three‐dimensional morphology of scaffolds while reducing fiber diameter. Furthermore, SEM images and ATR‐FTIR results confirmed that SPIONs and Si‐SPIONs‐were coated on scaffolds. A cytocompatibility assay showed a non‐toxic effect on MG‐63 osteoblast‐like cells in all scaffolds. Additionally, higher MC3T3‐E1 pre‐osteoblastic cell adhesion efficiency and proliferation are achieved for the alkaline‐treated scaffolds and SPIONs/Si‐SPIONs‐coated scaffolds. All samples demonstrated the ability to generate heat, useful for hyperthermia‐treatment, when subjected to an alternating magnetic field. Overall, the findings suggest that the strategy of coating PCL‐MEW scaffolds with SPIONs/Si‐SPIONs has the potential to improve scaffold performance for biomedical applications, especially for producing magnetically responsive MEW scaffolds.

## Graphical abstract

Polycaprolactone is used to fabricate superparamagnetic iron oxide nanoparticle (SPION) and silica‐coated‐SPION (Si‐SPION)‐coated scaffolds by melt electrowriting (MEW). The scaffolds’ hydrophilicity is modified by alkaline treatment. Scaffolds exhibit non‐toxicity. High pre‐osteoblastic (MC3T3‐E1) cell adhesion and proliferation are achieved, while all scaffolds show the ability to generate heat, useful for hyperthermia treatment when subjected to an alternating magnetic field.

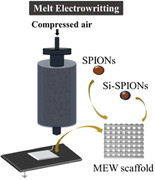

## 1 Introduction

Melt electrowriting (MEW) is a fast‐emerging additive manufacturing technique based on a combination of electrospinning and three‐dimensional (3D) melt extrusion printing methods.^[^ ^1^ ^]^ During the MEW processing, designed geometries by computer software are deposited on the collector layer‐by‐layer by applying pneumatic extrusion and voltage to the extruded polymer melt, thus, obtaining highly‐ordered microfibrous scaffolds with the fiber diameter ranging from ≈0.8 to ≈50 microns.^[^ ^1^, ^2^ ^]^ Over the last decades, polycaprolactone (PCL) has been commonly used to fabricate these highly ordered MEW scaffolds.^[^ ^2^, ^3^ ^]^ Previous studies demonstrated that PCL is a promising aliphatic thermoplastic polyester for MEW due to its excellent processability, biocompatibility, and high mechanical properties.^[^ ^3^, ^4^ ^]^ However, PCL's biodegradation, hydrophilicity, cell adhesion, and proliferation responses are limited.^[^ ^5^ ^]^ To overcome this drawback, researchers have recently focused on alkaline surface treatment, a simple and effective strategy to enhance hydrophilicity and cell affinity by breaking the ester bond of PCL, allowing carboxyl and hydroxy group formation on the surface.^[^ ^6^ ^]^ For instance, Muerza‐Cascante et al.^[^ ^7^ ^]^ found that primary human osteoblast and placenta‐derived mesenchymal stem cell attachment on PCL‐MEW scaffolds improved after alkaline treatment.^[^ ^7^ ^]^ Similar results were reported by Meng et al.,^[^ ^8^ ^]^ who showed that the alkaline treatment effect on poly (L‐lactic acid)‐MEW scaffolds enhanced the surface roughness, mechanical behavior as well as immature bone tissue amount.^[^ ^8^ ^]^

Magnetic nanoparticles are one of the interesting nanomaterials in biomedical applications such as cancer treatment, targeted drug delivery, and magnetic particle imaging due to their minimal toxicity, stability, biocompatibility, and controllable size.^[^ ^9^, ^10^, ^11^ ^]^ In particular, superparamagnetic iron oxide nanoparticles (SPIONs) incorporated into membranes,^[^ ^12^ ^]^ electrospun fibers,^[^ ^13^, ^14^ ^]^ and hydrogels^[^ ^15^ ^]^ have been widely studied. Recently, magnetically responsive MEW scaffolds have been gaining attention. For instance, Mueller et al.^[^ ^16^ ^]^ investigated ultrasmall SPION‐containing PCL‐MEW scaffolds to improve visibility in non‐invasive magnetic resonance imaging.^[^ ^16^ ^]^ In another study, Saiz et al.^[^ ^17^ ^]^ focused on designing a four‐dimensional (4D) printing technique using iron‐oxide nanoparticles' magnetic response in PCL‐MEW scaffolds.^[^ ^17^ ^]^ A similar result was reported by Kade et al.,^[^ ^18^ ^]^ who produced stimuli‐responsive 4D printed scaffolds based on carbonyl iron particles containing poly (vinylidene fluoride).^[^ ^18^ ^]^

The aim of this study was to evaluate PCL‐MEW scaffolds coated with SPIONs for biomedical applications. In this regard, PCL‐MEW scaffolds were produced and then modified with an alkaline surface treatment to improve the scaffold's hydrophilicity and to facilitate the coating with SPIONs. Afterward, SPIONs and silica‐coated‐SPIONs (Si‐SPIONs) produced according to our previous study^[^ ^10^ ^]^ were coated into MEW scaffolds. Lastly, we investigated the SPIONs or Si‐SPIONs‐coated MEW scaffolds in terms of physical, chemical and biological activities such as 2,2‐diphenyl‐1‐picrylhydrazyl (DPPH) radical scavenging activity, antibacterial property, and cytocompatibility.

## 2 Results and Discussion

### 2.1 Optimization of MEW Parameter

MEW is a multi‐parametric method in which different processing parameters, such as applied air pressure, voltage, printing speed, temperature, and nozzle‐to‐collector distance, significantly affect the printing outcomes, including stable printing conditions and uniformity of fibers. Optimizing these parameters is fundamental to achieving improved printing results. During MEW processing, the applied air pressure influences the material`s flow and can lead to the unstable formation of Taylor cone and jet, and non‐uniform fiber deposition. In this study, firstly, to study the dependency of pressure on MEW scaffold morphology, other parameters such as temperature (85 °C), nozzle‐to‐collector distance (1 mm), voltage (4 kV), and translation speed (25 mm^−1^ s) were kept constant, while the pressure was varied (200, 220, 240, and 255 kPa). Light microscopy images showed that the homogeneity and uniformity of the deposited PCL fibers increased with pressure. It could be due to the unstable jet formation, resulting in fiber pulsing when the pressure was below 255 kPa. In a related study, Hrynevich et al.^[^ ^19^ ^]^ investigated the effect of applied air pressure (from 0.5 to 4 bar) on PCL fiber morphology. The authors reported that the accurate placement of fiber and the straight fiber formation are adversely affected by applied air pressures below 0.5 bar.^[^ ^19^ ^]^

**Figure 1:**
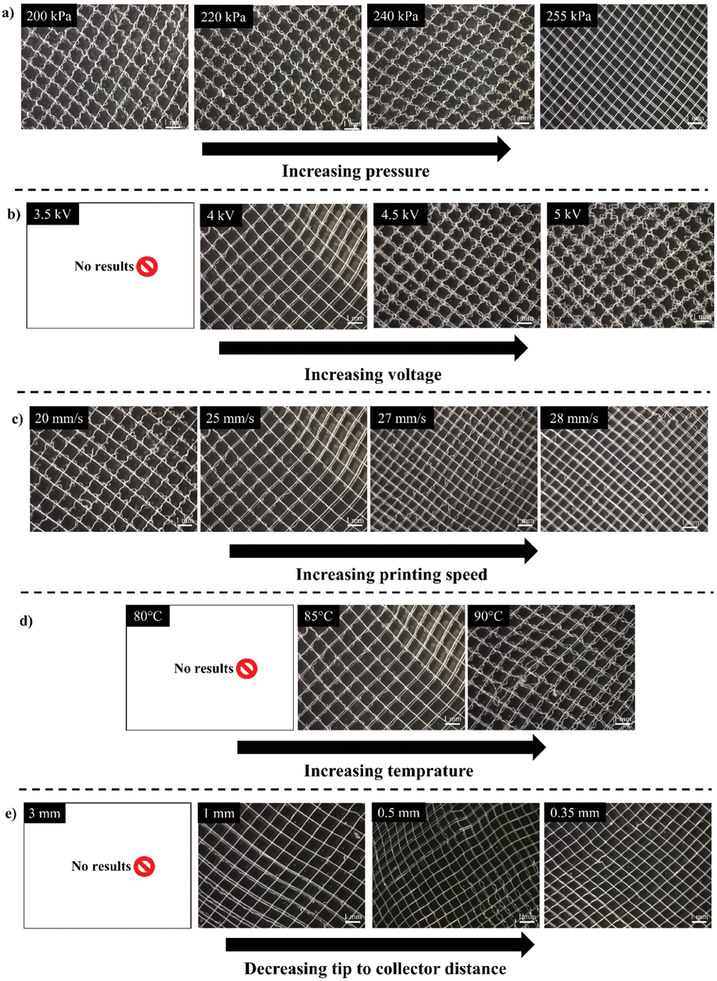
Representation of light microscope images of MEW scaffolds obtained with various parameters such as: a) Pressure (200, 220, 240, and 255 kPa), b) voltage (3.5, 4, 4.5, and 5 kV), c) printing speed (20, 25, 27, and 28 mm^−1^ s), d) cartridge temperature (80, 85, and 90 °C), and e) distance between nozzle to collector (1, 0.5, and 0.35 mm).

Additionally, temperature (85°C), nozzle‐to‐collector distance (1 mm), pressure (255 kPa), and printing speed (25 mm^−1^ s) were fixed to investigate the effect of the different voltages of 3.5, 4, 4.5, and 5 kV on the morphology of MEW scaffolds and fiber deposition. The light microscope images of PCL‐MEW scaffolds at different voltage configurations are depicted in **Figure** **1**b. The results revealed that the applied voltage of 3.5 kV was insufficient for fabricating scaffolds by MEW. Additionally, when the voltage increased from 4 to 5 kV, homogeneous fiber deposition significantly became unstable, indicating that the applied voltage was set over a threshold. This phenomenon could be attributed to the fact that increasing the voltage could change the jet pulsing and jet landing speed during the fibers pilling up; thus, the accumulated charge within the top layers of scaffolds may result in random fiber placement by attracting or repelling the next landing jet.^[^ ^20^ ^]^ By contrast, fibers printed at 4 kV showed improved uniformity. In a similar study, Cao et al.^[^ ^21^ ^]^ developed a method for tracking jet lag in real time to study the voltage influence on fiber uniformity. Their results suggested that increasing the voltage could lead to values over the threshold level, resulting in random jet lag fluctuation. In another study, Tourlomousis et al.^[^ ^22^ ^]^ reported that decreasing the voltage from 12.5 to 11.5 kV prevented unstable fiber deposition and yielded stable cone‐jet formation. In a recent study, Lu et al.^[^ ^23^ ^]^ fabricated PCL‐MEW scaffolds using different voltage configurations, including (+4 kV/0), (+3 kV/−1 kV), (+2 kV/−2 kV),(+1 kV/−3 kV), and (0/−4 kV). The authors suggested that the fiber deposition was homogenous at (+4 kV/0) and (0/‐4 kV) with 200 and 500 µm fill in the distance, which is in agreement with the present study.

Next, the MEW scaffolds were fabricated by varying the printing speed (20, 25, 27, and 28 mm ^−1^s) to focus on the optimum value for stable and desired fiber deposition. For these experiments, temperature (85 °C), nozzle‐to‐collector distance (1 mm), pressure (255 kPa), and voltage (4 kV) were kept constant. The light microscopy image in Figure 1c shows random fiber deposition at a 20 mm^−1^ s speed, which might be due to the fiber jet buckling during low printing speeds because of inner longitudinal compression.^[^ ^21^, ^24^ ^]^ When the printing speed increased to 28 mm^−1^ s, the fiber jet was collected as a straight line directed to the collector. This result could be explained by the balance between inner longitudinal compression and tensile drag when the printing speed reaches a threshold value.^[^ ^21^, ^24^ ^]^ In a similar study, Hrynevich et al.^[^ ^19^ ^]^ reported that in PCL‐MEW scaffold the polymer and processing parameters influence continuous jetting. Their results indicated that raising the collector speed from 300 to 360 mm min^−1^, led to a higher collector speed than the critical translation speed (CTS), achieving straight fiber formation.^[^ ^19^ ^]^ In another study, various printing speeds (from 2 to 117 mm^−1^ s) effect on PCL‐MEW scaffold fabrication was evaluated by Tourlomousis et al.^[^ ^22^ ^]^ Their results indicated that fibers were randomly oriented at low speeds (2–8 mm^−1^ s). However, the authors stated that when increasing the printing speed to 83 mm^−1^ s, the fibers were well‐aligned with an average fiber diameter of 23 ± 1.5 µm.^[^ ^22^ ^]^

Furthermore, the temperature is another crucial parameter for optimizing the MEW scaffold printing process. The increasing temperature in the cartridge can change the polymer properties, such as polymer melt viscosity; thereby, the melt flow resistance through the nozzle declined due to the polymer melt viscosity`s reduction.^[^ ^3^, ^22^ ^]^ In contrast, low polymer melt temperature can reduce the fiber fusion in the nozzle and requires high pressure during the printing process.^[^ ^3^, ^22^ ^]^ Therefore, in this study, different cartridge temperatures of 80, 85, and 90 °C were compared regarding the PCL‐MEW scaffold fabrication. Throughout the experiment, the pressure (255 kPa), nozzle‐to‐collector distance (1 mm), printing speed (28 mm^−1^ s), and voltage (4 kV) were kept constant. As shown in Figure 1d, the MEW scaffold could not be handled, and the design stability was insufficient due to the lack of fusion of fibers when the cartridge temperature was set to 80 °C. At polymer melt temperatures of 85 °C (Figure 1d), the formation of fibers was influenced by the increasing temperature in the cartridge as the heat could increase the viscosity of the polymer melt. Additionally, increasing the polymer melt temperature to 90 °C led to nonuniform fiber formation because of the volumetric flow rate rising inside the nozzle. Similar results were recently reported by Warren et al.,^[^ ^25^ ^]^ who produced square and rectangular macroscale geometries of PCL‐MEW scaffolds under different temperature conditions (70, 80, and 90 °C). The author noted that increasing the melt temperature affected the fiber formation, diameter, and interfiber spacing for both square and rectangular scaffolds.^[^ ^25^ ^]^

Finally, at the constant parameters: temperature (85 °C), pressure (255 kPa), printing speed (28 mm^−1^ s), and voltage (4 kV), the influence of nozzle to collector distance was investigated, considering the following values: 3, 1, 0.5, and 0.35 mm. The nozzle‐to‐collector distance is a fundamental parameter to comprehend the fiber formation, which is also correlated with voltage, pressure, viscosity, surface tension, and gravitational forces.^[^ ^20^, ^22^ ^]^ During the printing process, there is no change in the droplets at the nozzle tip if the gravitational force is less than the surface tension.^[^ ^4^, ^24^ ^]^ Then, an electric field is applied between the nozzle and the collector by a high voltage supply; thus, increasing the electric field intensity also increases proportionally the liquid surface, resulting in a Taylor cone‐shaped droplet.^[^ ^4^, ^24^ ^]^ Accordingly, if the nozzle‐to‐collector distance increases, the fiber diameter increases due to the electrical field intensity decline.^[^ ^4^, ^24^ ^]^ Figure 1e shows light microscopy images of PCL‐MEW scaffolds printed with three different nozzle‐to‐collector distances. The MEW scaffold's design stability was inadequate at the highest nozzle‐to‐collector distance (3 mm). The results indicated that decreasing the nozzle‐to‐collector distance improved straight fiber formation with desired geometry. In a similar study, Tourlomousis et al.^[^ ^22^ ^]^ reported that reducing the distance between the nozzle and collector from 15 to 10 mm facilitates excess material`s stretching collected at the tip owing to higher electrical field intensity.Additionally, the authors argued that only reducing the distance is not sufficient to obtain straight fiber formation; besides, equilibrium conditions are also required, provided with polymer flow rate and applied voltage.^[^ ^22^ ^]^

**Figure 2:**
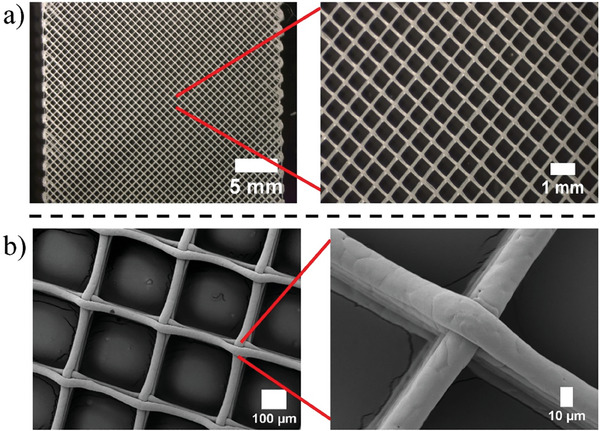
Optimized PCL‐MEW scaffold: a) Light microscopy images at two magnifications and b) Scanning electron microscopy (SEM) images. Parameters: 255 kPa of pressure, 4 kV of voltage, 28 mm^−1^ s of printing speed, 85 °C of polymer melt temperature, and 0.35 mm of nozzle‐collector distance.

As a result, the combination of printing parameters can affect straight fiber formation and the desired geometry of PCL‐MEW scaffolds. In the present investigation, the optimal values were obtained at 255 kPa pressure, 4 kV voltage, 28 mm^−1^ s printing speed, 85 °C polymer melt temperature, and 0.35 mm nozzle‐collector distance (**Figure** **2**).

### 2.2 Optimization of Surface Modification

PCL is the most widely used thermoplastic polyester for MEW technology because of its excellent processability, biocompatibility, and high mechanical properties.^[^ ^1^ ^]^ Nevertheless, PCL is limited by low biodegradability, low wettability, and a lack of active sites for biomolecule immobilization.^[^ ^5^ ^]^ In a related study, Muerza‐Cascante et al.^[^ ^7^ ^]^ reported that alkaline treatment enhances primary human osteoblast and placenta‐derived mesenchymal stem cell attachment on PCL‐MEW scaffolds. Additionally, the authors suggested that the alkaline treatment, which breaks the ester link in PCL to form carboxyl and hydroxyl groups, could improve the calcium‐phosphate coating of PCL‐MEW scaffolds due to these active groups.^[^ ^7^ ^]^ The present study modified PCL‐MEW scaffolds by alkaline treatment to increase hydrophilicity, cell affinity, and coating potential with SPIONs. In this regard, various surface treatment times (1, 2, and 3 h) were investigated regarding surface morphology, surface chemistry, and the presence of hydrogen (H^+^) ions.

**Figure 3:**
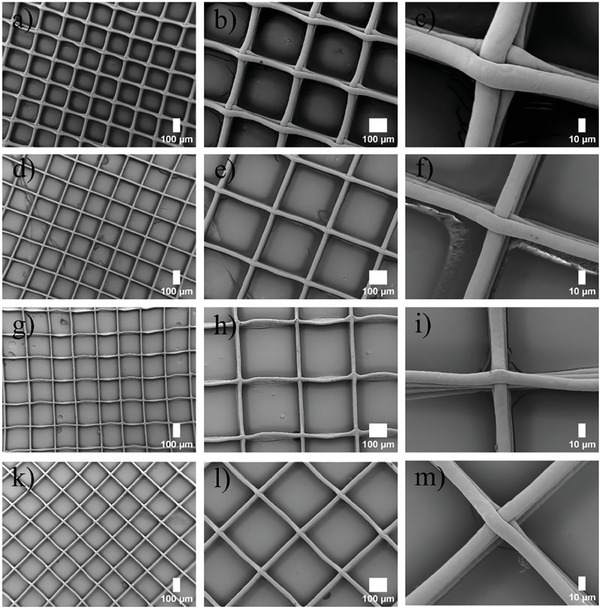
SEM images of scaffolds before and after alkaline treatment: a–c) PCL, d–f) PCL‐1 h NaOH, g–i) PCL‐2 h NaOH, and k–m) PCL‐3 h NaOH.

**Figure** **3** depicts alkaline treated and untreated PCL‐MEW scaffolds, respectively. The SEM images revealed that the morphology of the PCL‐MEW scaffolds was not affected by the alkaline treatment, whereas increasing immersion time reduced the average fiber diameters (**Table** **1**). In a recent study, Meng et al.^[^ ^8^ ^]^ investigated the alkaline treatment effect on poly (L‐lactic acid)‐MEW scaffolds. Their results demonstrated that the alkaline treatment enhanced the surface roughness, mechanical behavior, and immature bone tissue amount.^[^ ^8^ ^]^ Additionally, as given in Table 1, although the average pore size of the scaffolds was increased after alkaline treatment, it has no significant difference between the different treatment times. In a related study, Qui et al.^[^ ^26^ ^]^ reported that the PCL scaffold`s pore size increased after alkaline treatment,^[^ ^26^ ^]^ which is in line with our results.

**Table 1:** Average fiber diameter, and pore size of PCL‐MEW scaffolds before and after alkaline treatment.

Sample Code	Fiber diameter [µm]	Pore size [µm]		
		Average	*X*‐axis	*Y*‐axis
PCL	31 ± 3	278 ± 12	283 ± 12	274 ± 10
PCL‐1 h NaOH	23 ± 2	290 ± 10	292 ± 10	287 ± 8
PCL‐2 h NaOH	19 ± 4	300 ± 14	300 ± 17	300 ± 11
PCL‐3 h NaOH	13 ± 1	296 ± 17	295 ± 15	298 ± 17

**Figure 4:**
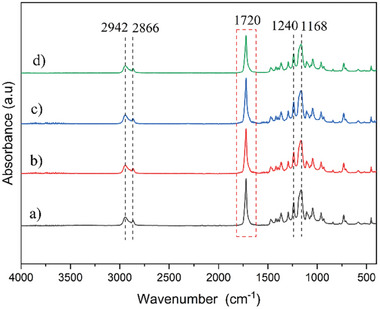
ATR‐FTIR spectra of PCL‐MEW scaffolds before and after alkaline treatment: a) PCL, b) PCL‐1 h NaOH, c) PCL‐2 h NaOH, and d) PCL‐3 h NaOH. The relevant peaks are discussed in the text.

On the other hand, attenuated total reflectance (ATR)‐Fourier‐transformed infarared spectroscopy (FTIR) analysis of the untreated and treated PCL‐MEW scaffolds was carried out to verify the effectiveness of the alkaline treatment, as shown in **Figure** **4**. The typical PCL peaks were at 2942 and 2866 cm^−1^, corresponding to the aliphatic groups (C–H).^[^ ^27^ ^]^ Further peaks were at 1720, 1240, and 1168 cm^−1^, which are related to a carbonyl group (C=O), C–O–C bond asymmetric, and symmetric stretching, respectively.^[^ ^28^ ^]^ Additionally, contrary to Takahiro Yew et al.,^[^ ^29^ ^]^ who investigated the alkaline hydrolysis of PCL electrospun nanofibers, in this study, a significant reduction in characteristic PCL peaks' intensity was not observed after alkaline treatment.

**Figure 5:**
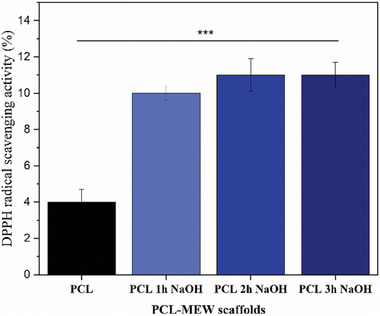
DPPH radical scavenging activity of PCL‐MEW scaffolds before and after surface modification. The value marked with asterisk (***) was significantly different based on ANOVA with Bonferroni's test at *p* < 0.001.

Moreover, after alkaline treatment, the presence of hydrogen (H^+^) ions was investigated by a slightly modified DPPH radical scavenging activity assay, which is commonly used to quantify antioxidant activity. The principle of this assay is based on hydrogen donation or radical scavenging ability^[^ ^30^ ^]^ that could aid in investigating the hydrogen (H^+^) ions on the surface of PCL‐MEW scaffolds; thus, new polar groups, such as carboxyl and hydroxyl groups present after the alkaline treatment^[^ ^6^ ^]^ might be examined. As represented in **Figure** **5**, the results showed that the alkaline treatment enhanced the formation of oxygen functional groups compared to untreated PCL‐MEW scaffolds. In contrast, no significant difference was obtained for different treatment time points.

In conclusion, the PCL‐3 h NaOH scaffold was selected for further analysis based on its surface morphology, the results of the ATR‐FTIR analysis, and the presence of H^+^ ions.

### 2.3 Optimization of SPION Coating

**Figure 6:**
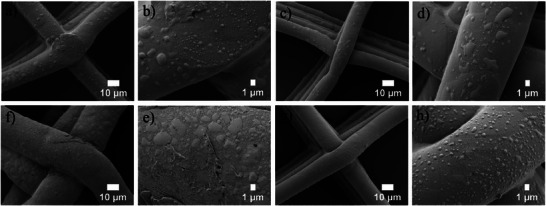
SEM images of PCL‐MEW scaffolds after coating with SPIONs and Si‐SPIONs: a,b) PCL 1 min SPIONs, c,d) PCL 3 min SPIONs, e,f) PCL 1 min Si‐SPIONs, and g,h) PCL 3 min Si‐SPIONs.

Recently, literature has been increasingly reporting magnetically responsive MEW scaffolds developed with magnetic nanoparticles.^[^ ^16^, ^17^ ^]^ Although magnetic nanoparticles are well‐known for magnetic resonance imaging, drug delivery, hyperthermia, transfections, and in vivo cell tracking applications owing to their small dimensions, high volume‐to‐surface ratio, and display of magnetic properties, their cytotoxicity depends on the nanoparticle`s size, surface, and dose.^[^ ^31^, ^32^ ^]^ Therefore, recent studies have focused on developments related to nanoparticle functionalization, such as silica shell on magnetic nanoparticles.^[^ ^10^, ^33^, ^34^ ^]^ In our previous study,^[^ ^10^ ^]^ TEM images of SPIONs illustrated that particles have a spherical shape with a diameter of about 10–15 nm; in addition, the particles exhibit a surface coating (SiO_2_) of 1–2 nm thickness. In the present study, **Figure** **6** shows the morphology of MEW scaffolds altered with SPIONs and Si‐SPIONs. The SEM images revealed that the morphology of MEW scaffolds slightly changed after 1 and 3 min coating, showing that the scaffolds had an open‐pore structure and rough surface. In a related study, Hu et al.^[^ ^35^ ^]^ reported that magnetic iron oxide nanoparticles‐containing PCL fibers showed agglomerated morphology on the fiber`s surface, which is in agreement with our results.^[^ ^35^ ^]^

**Figure 7:**
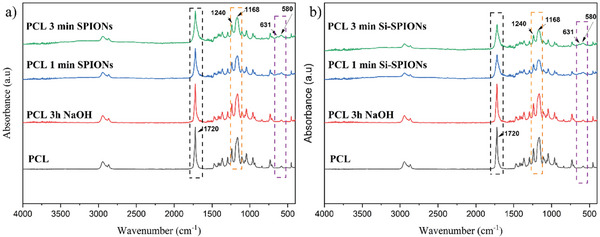
ATR‐FTIR spectra of PCL‐MEW scaffold: after coating with a) SPIONs and b) Si‐SPIONs.

Additionally, after coating for 1 and 3 min, the presence of SPIONs or Si‐SPIONs was analyzed by ATR‐FTIR spectroscopy. As shown in **Figure** **7**a,b, the characteristic peaks of PCL were observed at 1720, 1240, and 1168 cm^−1^, respectively, related to a carbonyl group (C=O), C–O–C bond asymmetric, and symmetric stretching.^[^ ^36^ ^]^ The IR spectrum of SPIONs exhibits bands at 600–500 and 575–550 cm^−1^, corresponding to M_T_–O–M_O_ and Fe–O stretching, respectively.^[^ ^37^ ^]^ As presented in Figure 7, the intensity of typical PCL peaks was reduced after the coating with SPIONs or Si‐SPIONs. Additionally, peaks attributed to SPIONs were visible after the coating process. However, the symmetric stretching band of ‐Si‐O‐Si‐ at 800 cm^−1[^ ^38^ ^]^ could not be detected for Si‐SPIONs coated scaffolds, which could be attributed to inhomogeneous Si‐SPIONs coating on MEW scaffolds as well as the thin Si‐layer of Si‐SPIONs.^[^ ^10^ ^]^ As a result, the analysis of the MEW scaffolds showed specific PCL and SPION peaks, proving the interaction between PCL and SPIONs.

**Figure 8:**
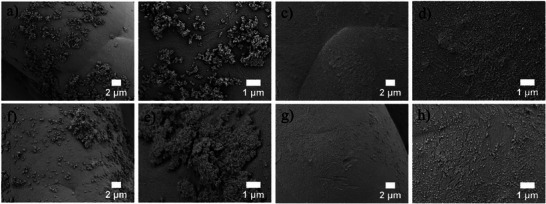
SEM images of SPIONs or Si‐SPIONs coated PCL‐MEW scaffolds after immersion in DMEM for 7d: a,b) PCL 1 min SPIONs, c,d) PCL 3 min SPIONs, e,f) PCL 1 min Si‐SPIONs, and g,h) PCL 3 min Si‐SPIONs.

On the other hand, the coating stability of SPIONs or Si‐SPIONs on the MEW scaffolds was investigated by soaking in Dulbecco´s Modified Eagle´s medium (DMEM) for 7 days (**Figure** **8**). SEM images revealed that after a 7‐day immersion, SPIONs and Si‐SPIONs coating promoted the nucleation of apatite‐like minerals. Our previous results^[^ ^10^ ^]^ showed that although SPIONs are positively charged in citric acid they become negatively charged, which could explain this phenomenon.

Based on surface morphology and ATR‐FTIR findings, a coating time of 3 min for SPIONs or Si‐SPIONs was selected for further investigation.

### 2.4 Calorimetric Test

**Figure 9:**
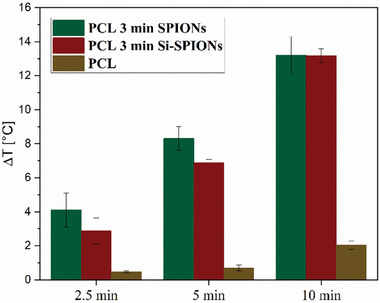
Calorimetric test under alternating magnetic field for SPIONs and Si‐SPIONs coated MEW scaffolds.

In our previous study,^[^ ^10^ ^]^ the magnetic properties of SPIONs and Si‐SPIONs were evaluated using a vibrating samples magnetometer (VSM‐Lakeshore). The results indicated that SPIONs and Si‐SPIONs exhibited a superparamagnetic behavior since there was no evidence of remnant magnetization and coercivity. Based on this information, this study focused on the ability of the SPIONs and Si‐SPIONs‐coated MEW scaffolds to release a quantity of heat useful for killing tumor cells with hyperthermia by using an induction furnace. **Figure** **9** shows the temperature increase (ΔT) of PCL, SPIONs, and Si‐SPIONs‐coated MEW scaffolds subjected to alternating magnetic fields. Each sample type showed an increase in temperature over time, while SPIONs and Si‐SPIONs‐coated MEW scaffolds displayed higher temperature increase than PCL‐MEW scaffolds, through Néel and Brownian relaxations. In particular, SPIONs‐coated MEW scaffolds reached slightly higher temperatures than Si‐SPIONs‐coated ones during the first minutes, probably due to the presence of the silica shell, while after 10 min no differences were observed between the scaffolds. In any case, in both cases (SPIONs and Si‐SPIONs‐coated MEW scaffolds), the obtained increase in temperature is widely useful for magnetic hyperthermia therapy of tumors.^[^ ^39^ ^]^

### 2.5 DPPH Radical Scavenging Activity

**Figure 10:**
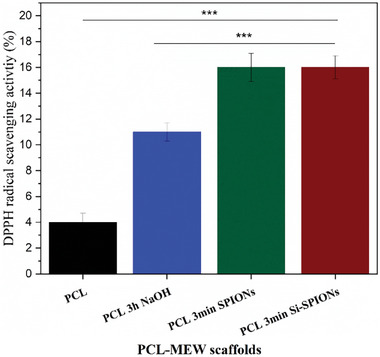
Antioxidant activity of PCL‐MEW scaffolds. When analyzed by Bonferroni's test, the asterisks indicate a significant difference (***p < 0.001).

Iron oxide nanoparticles could trigger the reduction of Fe (III) to Fe (II) in Fenton's reaction and affect intracellular oxidation‐reduction reactions, which can lead to oxidative toxicity due to excess reactive oxygen species (ROS).^[^ ^40^, ^41^, ^42^ ^]^ Previous reports have demonstrated that iron oxide nanoparticles induce ROS, resulting in cancer cell death.^[^ ^42^, ^43^ ^]^ For instance, Khan et al.^[^ ^42^ ^]^ investigated the iron oxide nanoparticles' effect on human cancer cells and normal human lung fibroblast cells. Their results indicated that iron oxide nanoparticles induced ROS formation in a concentration‐dependent manner. Interestingly, the authors stated that iron oxide nanoparticles were cytotoxic to human cancer cells but did not harm normal human lung fibroblasts.^[^ ^42^ ^]^ Therefore, the present study investigated the DPPH radical scavenging activity of the SPIONs or Si‐SPIONs‐incorporated MEW scaffolds to understand hydrogen donation or radical scavenging ability. **Figure** **10** depicts that the DPPH radical scavenging activity of the scaffolds exhibited a remarkable increase after alkaline treatment. Additionally, the SPIONs or Si‐SPIONs coated scaffolds' DPPH radical scavenging activity was 16 ± 1. The results confirmed that coating SPIONs or Si‐SPIONs on MEW scaffolds improved the radical scavenging ability. However, there was no significant difference between SPIONs and Si‐SPIONs coated scaffolds.

### 2.6 Antibacterial Activity

**Figure 11:**
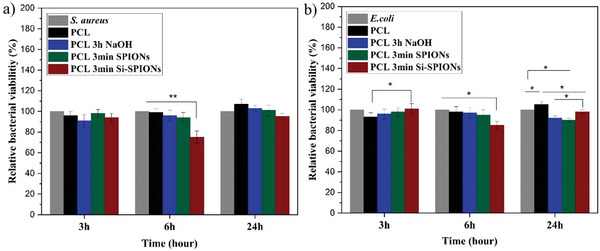
Antibacterial activity of PCL‐MEW scaffolds according to contact turbidity assay: a) *S. aureus* and b) *E. coli*. The asterisks indicate a significant difference (*p < 0.05, and **p < 0.01) when analyzed by Bonferroni's test.

It is well known that magnetic nanoparticles possess several advantages that make them ideal for biomedical applications, including inexpensive synthesis, feasibility to produce large quantities, biocompatibility, and environmental safety.^[^ ^44^ ^]^ Additionally, magnetic nanoparticles, such as magnetite (Fe_3_O_4_) and maghemite (γ‐Fe_2_O_3_), have been successfully used to inactivate various gram‐positive and gram‐negative bacteria.^[^ ^45^ ^]^ For instance, Prabhu et al.^[^ ^46^ ^]^ found that magnetic nanoparticles showed strong antibacterial properties against gram‐positive and gram‐negative bacteria when the concentration of Fe_3_O_4_ nanoparticles was increased from 20 to 150 µg ml^−1^.^[^ ^46^ ^]^ In another study, Chaurasia et al.^[^ ^41^ ^]^ investigated the positively charged magnetic core‐shell nanoparticles` bactericidal effect by electrostatic interaction. According to their results, *Escherichia coli* and *Staphylococcus aureus* bacteria can be completely inhibited within 30 min after exposure to the radiofrequency current due to the membrane losing its potential and the membrane‐associated complexes becoming dysfunctional.^[^ ^41^ ^]^ In the current study, the antibacterial activity of SPIONs and Si‐SPIONs‐coated PCL‐MEW scaffolds was tested with *S. aureus* and *E. coli* using contact turbidity assay, as illustrated in **Figure** **11**. The bacterial viability results showed that incorporating the nanoparticles, particularly Si‐SPIONs, improved the antibacterial effect of PCL‐MEW scaffolds against both *S. aureus* and *E. coli* bacteria strains for 6 h incubation. However, after 24 h incubation, scaffolds were ineffective for both bacteria strains. These results could be related to the SPIONs' surface morphology and charge.^[^ ^46^, ^47^ ^]^ For instance, SPIONs can inhibit the bacteria due to their small size (10–15 nm), penetrating the bacterial cell.^[^ ^47^ ^]^ On the other hand, SPIONs bear a positive charge while the bacterial cell wall is negatively charged; due to this, electromagnetic interaction; takes place, which can decompose the bacterial cell wall.^[^ ^46^ ^]^

Consequently, the findings in this study suggested that the Si‐SPIONs‐coated PCL‐MEW scaffolds can be exploited as antibacterial scaffolds.

### 2.7 In Vitro Cytotoxicity Assay

**Figure 12:**
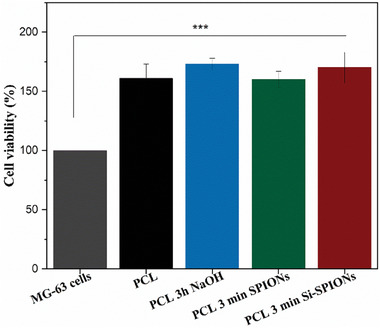
Viability of MG‐63 osteoblast‐like cells after exposure to PCL‐MEW scaffolds for 48 h. The value marked with asterisk (***) was significantly different based on ANOVA with Bonferroni's test at *p* < 0.001.

The prime objective of cancer treatment is targeting cancer cells without damaging normal cells.^[^ ^10^ ^]^ In this regard, SPIONs are commonly used in cancer treatment, particularly to treat bone cancer, due to their minimal toxicity, stability, biocompatibility, controllable size, and magnetic properties.^[^ ^48^, ^49^ ^]^ Herein, the effect of MEW scaffolds coated with SPIONs or Si‐SPIONs on MG‐63 osteoblast‐like cells was investigated using an indirect cytotoxicity assay. In this assay, PCL‐MEW scaffolds were placed onto cell strainers, immersed in fresh cell medium without being in contact with the MG‐63 osteoblast‐like cells, and the WST‐8 assay was used, as shown in **Figure** **12**. According to ISO‐10993‐5, cell viability above 80% is considered a non‐toxic material.^[^ ^50^ ^]^ As shown in Figure 12, all the PCL‐MEW scaffolds showed a non‐cytotoxic effect on MG‐63 osteoblast‐like cells. However, there is no statistically significant difference between the scaffolds. A similar result was recently reported by Shuai et al.,^[^ ^51^ ^]^ who investigated iron magnetic nanoparticles‐loaded poly‐L‐lactide/polyglycolic acid (PLLA/PGA) scaffolds produced by selective laser sintering. Their results indicated that incorporating iron magnetic nanoparticles was not toxic for MG‐63 osteoblast‐like cells. Additionally, the authors stated that magnetic scaffolds promoted proliferation and alkaline phosphatase activity.^[^ ^51^ ^]^ In another study, Kade et al.^[^ ^18^ ^]^ reported that poly (vinylidene fluoride) MEW scaffolds containing 1, 5, 15, and 30 wt% of carbonyl iron particles showed no adverse effect on murine fibroblast viability. Furthermore, the authors suggested that carbonyl iron particles‐contained MEW scaffolds can be used as magnetically active scaffolds in stimuli‐responsive applications.^[^ ^18^ ^]^

### 2.8 In Vitro *C*ell Proliferation

**Figure 13:**
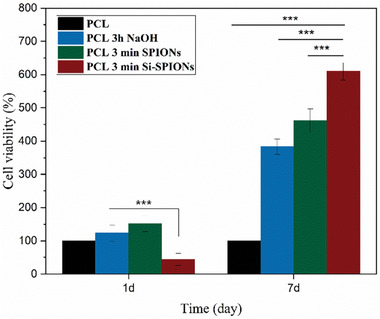
Viability of MC3T3‐E1 pre‐osteoblastic cells after seeded on the PCL‐MEW scaffolds for 1 and 7 days incubation period. Values with different asterisks (***) were significantly different based on ANOVA with Bonferroni's test at *p* < 0.001.

SPION's size, weight percentage, surface charge, and functionalization might affect the generation of reactive oxygen species, altering cell metabolism.^[^ ^40^, ^41^ ^]^ Previous studies have reported that iron oxide nanoparticles reduce human cancer cells' viability without adversely affecting other human cells.^[^ ^42^, ^52^ ^]^ The present study examined the effect of SPIONs or Si‐SPIONs coating on PCL‐MEW scaffolds on the MC3T3‐E1 pre‐osteoblastic cells' proliferation. In this regard, cells seeded on the MEW scaffolds were characterized with direct contact cell viability using WST‐8 colorimetric assay. **Figure** **13** shows the cell viability test of MC3T3‐E1 pre‐osteoblastic cells at 1 and 7 days. The results demonstrated that after 1‐day cultivation, Si‐SPIONs‐coated MEW scaffolds declined the MC3T3‐E1 pre‐osteoblastic cell viability compared to the uncoated PCL‐MEW scaffold. Following a 7‐day incubation period, the viability percentages of MC3T3‐E1 pre‐osteoblastic cells were dramatically increased for the SPIONs and Si‐SPIONs MEW scaffolds. Herein, it is important to highlight that incorporating SPIONs or Si‐SPIONs on MEW scaffolds enhanced cell viability compared to neat PCL and 3 min alkaline treated PCL‐MEW scaffolds, confirming that the SPIONs or Si‐SPIONs‐coated MEW scaffolds encourage adhesion of cells. Additionally, this result is consistent with research conducted by Mueller et al.,^[^ ^16^ ^]^ who fabricated MEW scaffolds incorporating ultrasmall SPIONs. The researchers stated that human umbilical artery smooth muscle cells proliferated and adhered to all scaffolds with excellent viability.^[^ ^16^ ^]^ In another study, Maleki‐Ghaleh et al.^[^ ^52^ ^]^ found that iron oxide nanoparticles in hydroxyapatite scaffolds enhanced the proliferation of human fibroblast cells.^[^ ^52^ ^]^ Similarly, Kim et al.^[^ ^53^ ^]^ indicated that magnetite nanoparticles‐incorporated PCL scaffolds showed tissue compatibility and new blood vessel formation 2 weeks after being implanted in rats.^[^ ^53^ ^]^ The findings in the present study demonstrated that the SPIONs or Si‐SPIONs‐coated MEW scaffolds exhibit promising properties that support their potential use in bone repair and regeneration.

### 2.9 Cell Morphology and Staining

**Figure 14:**
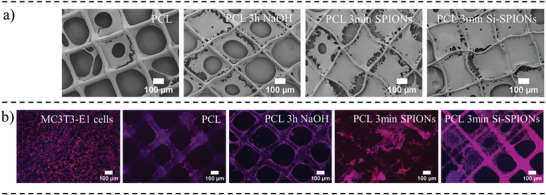
MC3T3‐E1 pre‐osteoblastic cell morphology after the 7‐day incubation: a) SEM images of MC3T3‐E1 pre‐osteoblastic cells on PCL‐MEW scaffolds, b) Fluorescence microscopy images of MC3T3‐E1 pre‐osteoblastic cells on PCL‐MEW scaffolds. F‐actin and nucleus stained red and blue, respectively.

The morphology of MC3T3‐E1 cells was evaluated using SEM analysis after a 7‐day incubation period to assess cell attachment and proliferation. SEM images in **Figure** **14**a show the behavior of MC3T3‐E1 cells seeded on the alkaline‐treated and SPIONs or Si‐SPIONs‐coated PCL‐MEW scaffolds indicating no adverse effects, as demonstrated by the WST‐8 colorimetric assay. The results confirm that the MC3T3‐E1 pre‐osteoblastic cells were firmly attached to the scaffold. The cytoskeleton seems to be elongated on the SPIONs or Si‐SPIONs‐coated scaffolds compared to the PCL‐MEW scaffolds, showing the alkaline treatment and the incorporation of SPIONs or Si‐SPIONs affect cell attachment and morphology. Similar results were recently found by Meng et al.,^[^ ^54^ ^]^ who enhanced the bioactivity and cell affinity of PLLA‐MEW scaffolds via alkaline‐treatment. The authors suggested that alkaline‐treatment (in 0.5 M NaOH, for 2 h) improved the hydrophilicity of the PLLA‐MEW scaffolds, resulting in improved KUSA‐A1 cell viability as well as osteoinductive ability. In another study, Zhang et al.^[^ ^55^ ^]^ reported that magnetic iron oxide nanoparticle‐loaded electrospun nanofibers enhanced the cell affinity of NIH 3T3 cells.

On the other hand, MC3T3‐E1 cell adhesion and cell phenotype on MEW scaffolds were investigated by fluorescence microscopy after being incubated for 7 days. Figure 14b shows the MC3T3‐E1 pre‐osteoblastic cell distribution on the surface, cell nuclei (DAPI, 4′,6‐diamidino‐2‐phenylindole), and F‐actin (rhodamine‐phalloidin) after 7 days of incubation. The images demonstrate that MC3T3‐E1 pre‐osteoblastic cells were attached, stretched, and elongated on the SPIONs and Si‐SPIONs‐coated MEW scaffolds rather than on the PCL‐MEW scaffold. Similarly, the fluorescent image results of Mueller et al.^[^ ^16^ ^]^ showed that human umbilical artery smooth muscle cells were attached to and well‐spread on the surface of different concentrations of SPIONs (0.1, 0.2, and 0.3% (w/w))‐loaded PCL‐MEW scaffolds.^[^ ^16^ ^]^ Overall, in the current study, the SEM and fluorescence images agree with the WST‐8 colorimetric cell viability assay, confirming the cytocompatibility of SPIONs or Si‐SPIONs‐coated MEW scaffolds with MC3T3‐E1 pre‐osteoblastic cells that present firm attachment and growth.

## 3 Conclusion

This study investigated the fabrication and characterization of SPIONs or Si‐SPIONs coated MEW scaffolds within three stages: 1) optimization of PCL‐MEW scaffolds by examining the relative effect of processing parameters, 2) modification of MEW scaffolds using alkaline surface treatment to enhance hydrophilicity and coating process, and 3) coating of MEW scaffolds with SPIONs or Si‐SPIONs. The results demonstrated that reproducible optimal MEW scaffolds were obtained at 255 kPa pressure, 4 kV voltage, 28 mm^−1^ s printing speed, 85 °C polymer melt temperature, and 0.35 mm nozzle‐collector distance. Additionally, SEM images revealed that alkaline surface treatment had no adverse effect on MEW scaffold morphology. Furthermore, SEM images and ATR‐FTIR analysis confirmed that SPIONs and Si‐SPIONs coating onto the MEW scaffolds was successful. Moreover, the DPPH radical scavenging assay demonstrated that coating SPIONs or Si‐SPIONs on MEW scaffolds improved their radical scavenging ability. On the other hand, MEW scaffolds coated with SPIONs or Si‐SPIONs enhanced the cellular activity of MG‐63 osteoblast‐like and MC3T3‐E1 pre‐osteoblastic cells compared to neat PCL. The preliminary physical, morphological, and biological results of SPIONs or Si‐SPIONs‐coated MEW scaffolds showed their potential as scaffolds for bone tissue engineering. The calorimetric evaluation showed that both SPIONs and Si‐SPIONs‐coated scaffolds are potentially suitable for hyperthermia applications. However, further investigations using vibrating sample magnetometry should be pursued to investigate further the magnetic properties of the scaffolds.

## 4 Experimental Section

### Materials

Polycaprolactone (PCL, Mn = 45 kDa, 704105), 2,2‐diphenyl‐1‐picrylhydrazyl (DPPH, D9132), WST‐8 (Cell counting kitting‐8, 96992), sodium hydroxide (NaOH, S5881), iron(II) chloride tetrahydrate (FeCl_2_ × 4.H_2_O, 1.03861), iron(III) chloride tetrahydrate (FeCl_3_ × 6.H_2_O, 8.03945), ammonium hydroxide (NH_4_OH, 221228), citric acid (CA, W230633), tetraethyl orthosilicate (TEOS, 86578), and fetal bovine serum (FBS, F2442) were ordered from Sigma Aldrich (Darmstadt, Germany). Dulbecco's phosphate‐buffered saline (PBS, no calcium, no magnesium, 10010023), Dulbecco's modified Eagle's medium (DMEM) (10313021), α‐minimum essential medium (α‐MEM, 22571038), penicillin/streptomycin (PS, 15140‐122), trypsin/EDTA (25200‐056), 4′,6‐diamidino‐2‐phenylindole (DAPI, 62247), rhodamine‐phalloidin (R415), and L‐glutamine (25030081) were obtained from Gibco Life Technologies, ThermoFisher Scientific (Schwerte, Germany). Luria/Miller agar (X969.1) and lysogeny broth medium (6673.1) were obtained from Carl Roth GmbH (Karlsruhe, Germany). *S. aureus* (ATCC25923) and *E. coli* (ATCC25922) bacteria strains, which are purchased from American type culture collection (ATCC), were used for the antibacterial activity assay. MG‐63 osteoblast‐like cell line (86051601‐1VL), and MC3T3‐E1 pre‐osteoblastic cell line (99072810‐1VL) were ordered from Sigma Aldrich (Darmstadt, Germany) for the biological activity assay.

### Preparation of SPIONs

SPIONs and Si‐SPIONs were synthesized using the chemical co‐precipitation method in an aqueous solution according to Borroni et al.^[^ ^10^ ^]^ Briefly, to obtain a stoichiometric ratio of Fe^2+^/Fe^3+^ of 1:2, appropriate amounts of FeCl_2_ × 4.H_2_O and FeCl_3_ × 6.H_2_O were mechanically mixed in bi‐distilled water (0.01 M). Then, the pH of the solution was adjusted to around 10 by dropping NH_4_OH dropwise, followed by the solution turning black. Afterward, the solution was placed in an ultrasound bath for 20 min to form SPIONs. After the magnetic separation of SPIONs, a bi‐distilled water wash was conducted three times. Subsequently, to enhance the dispersion of SPIONs, it was suspended in a 0.05 M solution of citric acid (CA). In the following steps, the suspension pH was adjusted to 5.2 by dropwise NH_4_OH and was incubated in an orbital shaker (KS 4000i control, IKA) at 150 rpm for 90 min. Following CA grafting, functionalized nanoparticles were washed with bi‐distilled water using an ultrafiltration device (Solvent Resistant Stirred Cells – Merck Millipore, Darmstadt, Germany), resuspended in bi‐distilled water, and adjusted at pH 10.1 to induce deprotonation of the third carboxylic group, allowing an optimal dispersion of nanoparticles. In order to obtain Si‐SPIONs, CA‐stabilized SPIONs were coated with a silica shell following the Stöber method, as described elsewhere.^[^ ^10^ ^]^ First, TEOS, ethanol, and water (with ethanol: water ratio of 1:1) were mixed and added to CA‐stabilized SPIONs for 3 h at 25 °C and 150 rpm. Finally, the Si‐SPIONs were re‐dispersed in water after being washed with bi‐distilled water using the ultrafiltration device. The complete characterization of the used SPIONs was reported elsewhere.^[^ ^10^ ^]^

### Fabrication of MEW scaffolds

MEW scaffolds were fabricated via three axes moveable bioplotter (type BioScaffolder 3.1, GeSIM, Großerkmannsdorf, Germany) equipped with a custom‐made heated collector. The geometry of the scaffold was designed via the “ScaffoldGenerator” software of the bioplotter. Briefly, polycaprolactone (Mn = 45 kDa) pellets were placed into a 10 mL thermal metal cartilage and heated for 30 min to melt the PCL before insertion into the MEW heating head. The MEW process variables, namely pressure (200, 220, 240, or 255 kPa), voltage (3.5, 4, 4.5, or 5 kV), printing speed (20, 25, 27, or 28 mm^−1^ s), melt extrusion temperature (80, 85 or 90 °C), and nozzle‐collector distance (3, 1, 0.5, and 0.35 mm), were investigated systematically at room temperature. A square wave pattern (13 × 13 mm) was used to fabricate MEW scaffolds with 325 µm inner distance, which were configured layer‐by‐layer (0°/90°) to form 12 layers.

### Surface Modification of MEW Scaffolds and Coating with SPIONs and Si‐SPIONs

**Figure 15:**
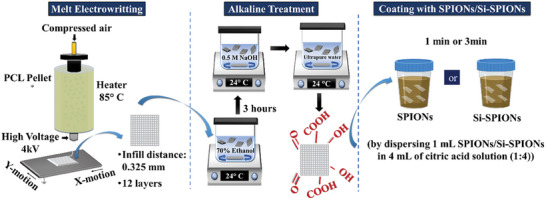
Schematic illustration of the melt electrowriting setup, alkaline treatment, and SPIONs‐coating process of PCL‐MEW scaffolds.

The MEW scaffold surface was modified by alkaline treatment assay to increase the hydrophilicity, according to Meng et al.,^[^ ^8^ ^]^ with slight changes. Briefly, the alkaline treatment was performed by soaking the MEW scaffolds in 70% ethanol solution for 15 min before washing with Milli Q water and etched to 0.5 M NaOH aqueous solution for various time points (1, 2, and 3 h, respectively). The scaffolds were thereafter rinsed several times at each time point in Milli Q water and, finally, dried at 37 °C for 24 h. After the alkaline treatment, MEW scaffolds were immersed into SPIONs and Si‐SPIONs solutions (by dispersing 1 mL SPIONs/Si‐SPIONs in 4 mL of CA solution [1:4]) for 1 or 3 min for each treatment time point, separately. Finally, scaffolds were left to dry at room temperature. The MEW scaffolds were labeled as PCL, PCL‐1 h NaOH, PCL‐2 h NaOH, PCL‐3 h NaOH, PCL 1 min SPIONs, PCL 3 min SPIONs, PCL 1 min Si‐SPIONs, and PCL 3 min Si‐SPIONs. An overview of the experimental approach is shown in **Figure** **15**.

### Characterization of MEW Scaffolds

The surface morphology of MEW scaffolds was analyzed by scanning electron microscopy (SEM, ETH: 2 kV, Everhart‐Thornley detector (SE2), AURIGA base 55, Carl Zeiss, Oberkochen, Germany). Before SEM analyses, the scaffolds were sputtered with a thin layer of gold using a sputter coater (Q150T Turbo‐Pumped Sputter Coater/Carbon Coater, Quorum Technologies). The average fiber diameter and pore size of MEW scaffolds were measured at 50 randomly selected points from the SEM images using the Image J analysis software (NIH, Bethesda, MD, USA). Additionally, the morphology of MEW scaffolds during the optimization of MEW parameters was investigated by light microscopy (Primo Vert, Carl Zeiss, Germany).

Attenuated total reflectance (ATR) – Fourier‐transformed infrared spectroscopy (FTIR) (IRAffinity‐1S Shimadzu) was used to identify the MEW scaffolds' functional groups. The infrared spectra were recorded with wavenumbers ranging between 400 and 4000 cm^−1^ at a spectral resolution of 4 cm^−1^ with 42 scans.

The structural stability of SPIONs or Si‐SPIONs‐coated MEW scaffolds was characterized in DMEM cell culture medium by 7 days immersion at 37 °C and 140 rpm. After 1 and 7 days of immersion, scaffolds were removed from the DMEM and rinsed three times with Milli Q water before drying at 37 °C overnight. Then, scaffolds were analyzed by SEM.

### Calorimetric Test

The ability of the SPIONs and Si‐SPIONs‐coated MEW scaffolds to generate heat was investigated using an induction furnace (Egma 6, FELMI Srl, Italy) at 6 kW and 219 kHz by measuring the increase of the temperature of a defined water volume (2 ml) up to 10 min using the set up described in.^[^ ^56^ ^]^ The temperature variation was calculated by measuring the temperature before and after the heating with a digital thermocouple. All the measurements were performed in triplicate.

### DPPH Radical Scavenging Activity

The DPPH radical scavenging assay described in the previous study^[^ ^57^ ^]^ was used to determine the radical scavenging ability of SPIONs, and Si‐SPIONs‐coated MEW scaffolds. Briefly, scaffolds were immersed overnight in a methanol solution of 2 ml. Following this, 2.5 mL of DPPH radical solution was mixed with 0.5 mL of methanol solution (at a concentration of 0.04 mg ml^−1^). After 90 min at room temperature in the dark, the absorbance was measured using UV–vis spectroscopy at 517 nm. Each sample was measured in triplicate. Methanol and DPPH radical solutions were used as blank and reference, respectively. The percentage of DPPH radical scavenging activity was achieved according to the following equation.

(1) $$DPPH\;radical\;scavenging\;activity\left(\%\right)=\frac{A_{control}-A_{sample}}{A_{control}}\times 100$$

### Antibacterial Activity

The antibacterial activity of MEW scaffolds was separately tested with *S. aureus* (Gram‐positive) and *E. coli* (Gram‐negative) bacteria, according to the previous study.^[^ ^27^ ^]^ The bacteria strains were inoculated in 10 mL of lysogeny broth medium at 37 °C overnight. Each bacterial suspension was then measured at 600 nm with an optical density (OD) device (Thermo ScientificTM GENESYS 30TM, Germany) to establish the absorbance value at 0.015 nm. After sterilization by UV irradiation for 1 h, the scaffolds were immersed in a lysogeny broth medium containing 20 µL of bacteria suspension. Subsequently, all the scaffolds were incubated at 37 °C for various times (3, 6, and 24 h). Finally, The OD values of the scaffolds were measured at 600 nm by a plate reader (PHOmo, Anthos Mikrosysteme GmbH, Germany), and the relative bacteria viability was calculated as follows.

(2) $$\begin{array}{c} Relative\;bacterial\;viability\left(\%\right)=\frac{Absorbance_{sample}-Absorbance_{blank}}{Absorbance_{Ref}-Absorbance_{blank}}\times 100 \end{array}$$

The lysogeny broth medium and bacterial suspensions were used as blank and control. All measurements were carried out in triplicate.

### In Vitro Cytotoxicity

The in vitro cytotoxicity of the MEW scaffolds was investigated by the WST‐8 cell proliferation assay kit (Sigma Aldrich, Germany) using the indirect contact method according to the ISO‐10993‐5 standard. Firstly, MG‐63 osteoblast‐like cells were cultured in DMEM supplemented with 10% (v/v) FBS, 1% (v/v) PS, and incubated at 37 °C in 5% CO_2_. Then, MG‐63 cells were seeded in 6‐well plates at a 4.5 × 10^5^ cells/well density and incubated for 24 h. Afterward, the sterilized scaffolds by UV irradiation for 1 h were placed onto the cell strainer, immersed in a fresh cell medium without touching the cells, and incubated for 48 h. After 48 h of incubation, the cell viability was analyzed by WST‐8 assay (1% (v/v), and the absorbance was measured at 450 nm by a microplate reader. The cell viability was calculated according to the following equation.

(3) $$Cellviability\left(\%\right)=\frac{Absorbance_{sample}-Absorbance_{blank}}{Absorbance_{Ref}-Absorbance_{blank}}\times 100$$

MG‐63 cells cultured without samples were used as a reference and labeled as “Ref,” the blank was WST‐8 solution. The results were normalized concerning a reference sample, and experiments were performed in triplicate.

### In Vitro Cell Proliferation

The in vitro cell proliferation of the MEW scaffolds was investigated by the WST‐8 cell proliferation assay kit (Sigma Aldrich, Germany) using the direct contact method according to the ISO‐10993‐5 standard. Firstly, MC3T3‐E1 pre‐osteoblastic cells were cultured in α‐ MEM supplemented with 10% FBS, 1% PS, and 1% L‐glutamine and incubated at 37 °C in 5% CO_2_. In parallel, the scaffolds with 13 mm in width × 13 mm in length were placed in 12‐well plates and sterilized under UV irradiation for 30 min on each side. Once MC3T3‐E1 pre‐osteoblastic cells reached 80% confluency, they seeded it onto the scaffolds at 2 × 10^5^ cells per well density. After seeding the cells, all MEW scaffolds were incubated at 37 °C in 5% CO_2_ for 1 and 7 days. Finally, the cell viability was analyzed by WST‐8 assay (5% (v/v), and the absorbance was measured at 450 nm by a microplate reader. The cell viability was calculated according to Equation (3). Native PCL scaffolds were used as a reference; the blank was a WST‐8 solution. The experiments were performed in triplicate.

### Cell Morphology and Fluorescence Staining

The morphology of the MC3T3‐E1 pre‐osteoblastic cells was characterized by SEM analysis after a 7‐day incubation. The scaffolds with cells were washed with PBS and were then fixed with SEM‐fixative‐I (0.2 M sodium cacodylate trihydrate, 0.1% [w/v] glutaraldehyde, 2% [w/v] paraformaldehyde, and 5% [w/v] sucrose) and SEM‐fixative‐II (0.2 M sodium cacodylate trihydrate, 0.3% (w/v) glutaraldehyde, and 2% [w/v]) for 1 h, respectively. Then, the scaffolds were rinsed with ethanol/water dilutions (from 30% to 100%) to dehydrate for 30 min and, finally, dried with a critical point dryer (EM CPD300, Leica, Germany).

The actin filaments and nuclei of the MC3T3‐E1 pre‐osteoblastic cells were separately stained by rhodamine‐phalloidin, and DAPI staining following the manufacturer's protocol. In brief, the cells were fixed with a 2.5% (v/v) paraformaldehyde. Subsequently, the cells were washed with PBS, followed by incubation of rhodamine‐phalloidin (8 µL in 1000 µL PBS) for 40 min at 37 °C. Then, the cells were stained with DAPI (1 µL in 1000 µL HBSS) for 5 min after being washed with PBS. Finally, fluorescence images were captured using fluorescence microscopy (DMI 6000B, Leica, Germany).

### Statistic Analysis

Data were analyzed by ANOVA and Bonferroni's test using the Origin (OriginLab, Northampton, MA, USA). The statistical significance level was performed at *p* < 0.05, *p* < 0.01, and *p* < 0.001. The results were denoted as mean ± SD.

## Conflict of Interest

The authors declare no conflict of interest.

## Author Contributions

I.U. conceived the original idea and carried out the conceptualization, methodology, validation, literature survey, formal analysis, investigation, data curation, visualization, and writing – of the initial draft. I.O. developed the methodology, validation, literature survey, formal analysis, investigation, data curation, visualization, and writing of the initial draft. M.M. provided supervision and conceptualization. E.V. planned the project, carried out supervision, and provided resources, project administration, and funding acquisition. A.R.B. conceived the original idea, planned the project, and carried out supervision, provided resources, project administration, and funding acquisition. All authors read and approved the final manuscript.

## Data Availability

The data that support the findings of this study are available from the corresponding author upon reasonable request.
